# Classifications of posterior malleolar fractures: a systematic literature review

**DOI:** 10.1007/s00402-022-04643-7

**Published:** 2022-12-05

**Authors:** Julia Terstegen, Hanneke Weel, Karl-Heinz Frosch, Tim Rolvien, Carsten Schlickewei, Elena Mueller

**Affiliations:** 1 Department of Trauma and Orthopaedic Surgery, University Medical Center Hamburg-Eppendorf, Martinistr. 52, 20246 Hamburg, Germany; 2 Department of Orthopedics, Bergman Clinics, Arnhem, The Netherlands; 3 Department of Trauma Surgery, Orthopaedics, and Sports Traumatology, BG Hospital Hamburg, Hamburg, Germany

**Keywords:** Ankle fracture, Posterior malleolus, Systematic literature review, Fracture classification

## Abstract

**Introduction:**

Complex ankle fractures frequently involve the posterior malleolus. Many classifications describing posterior malleolar fractures (PMF) exist. The aim of this study was to provide a systematic literature review to outline existing PMF classifications and estimate their accuracy.

**Methods:**

The databases PubMed and Scopus were searched without time limits. Only specific PMF classifications were included; general ankle and/or pilon fracture classifications were excluded. Selection and data extraction was performed by three independent observers. The systematic literature search was performed according to the current criteria of Preferred Reporting Items for Systematic Review and Meta-Analyses (PRISMA). The methodological quality of the included studies was quantified using the modified Coleman score.

**Results:**

A total of 110 studies with a total of 12.614 patients were included. Four main classifications were identified: Those describing the size of the posterior malleolar fracture (*n* = 66), Haraguchi (*n* = 44), Bartoníček/Rammelt (*n* = 21) and Mason (*n* = 12). The quality of the studies was moderate to good with a median Coleman-score of 43.5 (14–79) and a weighted median Coleman-score of 42.5 points. All classifications achieved a substantial to perfect score regarding the inter- and intraobserver reliability, with Mason scoring the lowest in comparison.

**Conclusions:**

None of the reviewed PMF classifications has been able to establish itself decisively in the literature. Most of the classifications are insufficient in terms of a derivable treatment algorithm or a prognosis with regard to outcome. However, as the Bartoníček/Rammelt classification has the greatest potential due to its treatment algorithm, its reliability in combination with consistent predictive values, its usage in clinical practice and research appears advisable.

**Supplementary Information:**

The online version contains supplementary material available at 10.1007/s00402-022-04643-7.

## Introduction

Ankle fractures present as one of the most common fractures with a prevalence of 4–9% [1, 2]. Posterior malleolar fracture (PMF), also known as malleolus tertius, posterior tibial fracture, or Volkmann-fragment appears in up to 44% of ankle fractures [3–5]. If the posterior malleolus is affected, therapy results may be worse and its presence in ankle fractures is known to be of negative prognostic value [1, 4, 6–10].

Decision-making to fixate PMF is still highly debatable and traditionally often based on fracture size measurement on radiographs, with lack of accuracy and poor reliability [11–18]. Nowadays, it is generally believed that the morphology of the fragment is more closely related to the fracture pattern and is, therefore, more important in classifying the fracture [14, 19–21]. Consequently, with regard to the proportion of the affected joint surface and recommendation for surgical fixation of PMF, there is a shift away from the 1/3 dogma [7, 17, 22–28]. With increasing understanding of fracture morphology and the routine use of computed tomography (CT), efforts have been made in recent years to establish new classification systems based on CT imaging [14, 29, 30]. Until now, there is no international consensus regarding classification and treatment of PMF [24, 31, 32].

A good classification system helps the orthopedic surgeon to identify and characterize a problem, suggest a potential prognosis, and offer guidance in determining the appropriate treatment method for a particular condition. To achieve optimal therapeutic results, a complete understanding of the morphology is indispensable.

Therefore, the aims of this systematic review were first, to determine how many studies use a classification of the PMF; second, to identify and to describe which classifications of PMF exist; third to examine which classification system does have the most reliable (inter- and intra- observer) scores; and fourth, to evaluate the predictive value of the classifications in terms of postoperative outcomes.

## Materials and methods

### Search strategy

The study protocol was registered in the PROSPERO database (CRD42021264268). The review was performed and reported according to the PRISMA 2020 checklist [33].

The electronic databases of the Cochrane Central Register of Controlled Trials, MEDLINE via PubMed and Scopus were searched systematically. The search was performed on the 20th of March 2021. The following search algorithm was used: (posterior AND ankle AND fracture) OR (posterior AND (malleolus OR malleolar) AND fracture) OR (ankle AND volkmann) OR (trimalleolar AND fracture) OR (posterior AND pilon AND fracture). A final update of the search was conducted 12th of May 2022 using the same search string. Furthermore, reference lists of relevant reviews and included articles were screened for additional articles. Bidirectional citation search was used including backward and forward citation search methods [34]. There were no limitations on journal or publication date of the article.

### Study selection

Studies reporting data on classification systems of trimalleolar ankle fractures were screened for using a PMF classification. Inclusion and exclusion criteria were cross checked by three reviewers (HW, JT, EM), first by screening the title and abstract, second by reading the full text. Clinical studies were included for data extraction. Cadaveric studies, review articles, case reports with fewer than 10 cases, studies that did not include a posterior malleolus specific classification, and studies not written in English, were excluded.

### Data extraction

The study selection and data extraction were independently performed by two review authors (JT, EM). Disagreements were discussed in a consensus meeting and if a disagreement persisted, a third reviewer (HW) made the final decision. Data were extracted from the included studies using a Microsoft Office^®^ Excel spreadsheet. This included the following data: study design, sample size and source, fragment characteristics (e.g., classification, displacement, treatment), reliability- and validity scores and additional data the classification addressed, like treatment allocation and prognostic value of it, were collected. Names of used classification system were listed and their frequency in use was counted.

### Study quality assessment

The methodological quality of the included studies was quantified using a modified Coleman score [35]. The modified Coleman score was applied by two independent reviewers (HW, JT) (Online Resource 1). The score is composed of two parts. Part A assesses study size, average follow-up time, percentage of patients with follow-up, number of interventions, study type, diagnostic certainty, description of surgical method, and postoperative rehabilitation. Part B is comprised of outcome criteria, procedure for assessing outcomes, and description of the subject selection process. The maximum score to be achieved is 100 points.

### Statistical analysis

The data were processed descriptively, therefore, no meta-analysis was performed. Patient demographic characteristics (number of patients/feet, patient age and sex) were summarized. Weighted median scores were calculated for the modified Coleman score and for the age of the evaluated patient cohort. Data analysis was performed using IBM SPSS Statistics Version 26.0 (IBM Corp., Armonk, NY, USA). The kappa values of inter- and intraobserver reliability were interpreted as defined by Landis and Koch (< 0.20: slight, 0.21–0.40: fair, 0.41–0.60: moderate, 0.61–0.80: substantial, 0.81–1.00: almost perfect) [36].

## Results

### Included studies

**Fig. 1:**
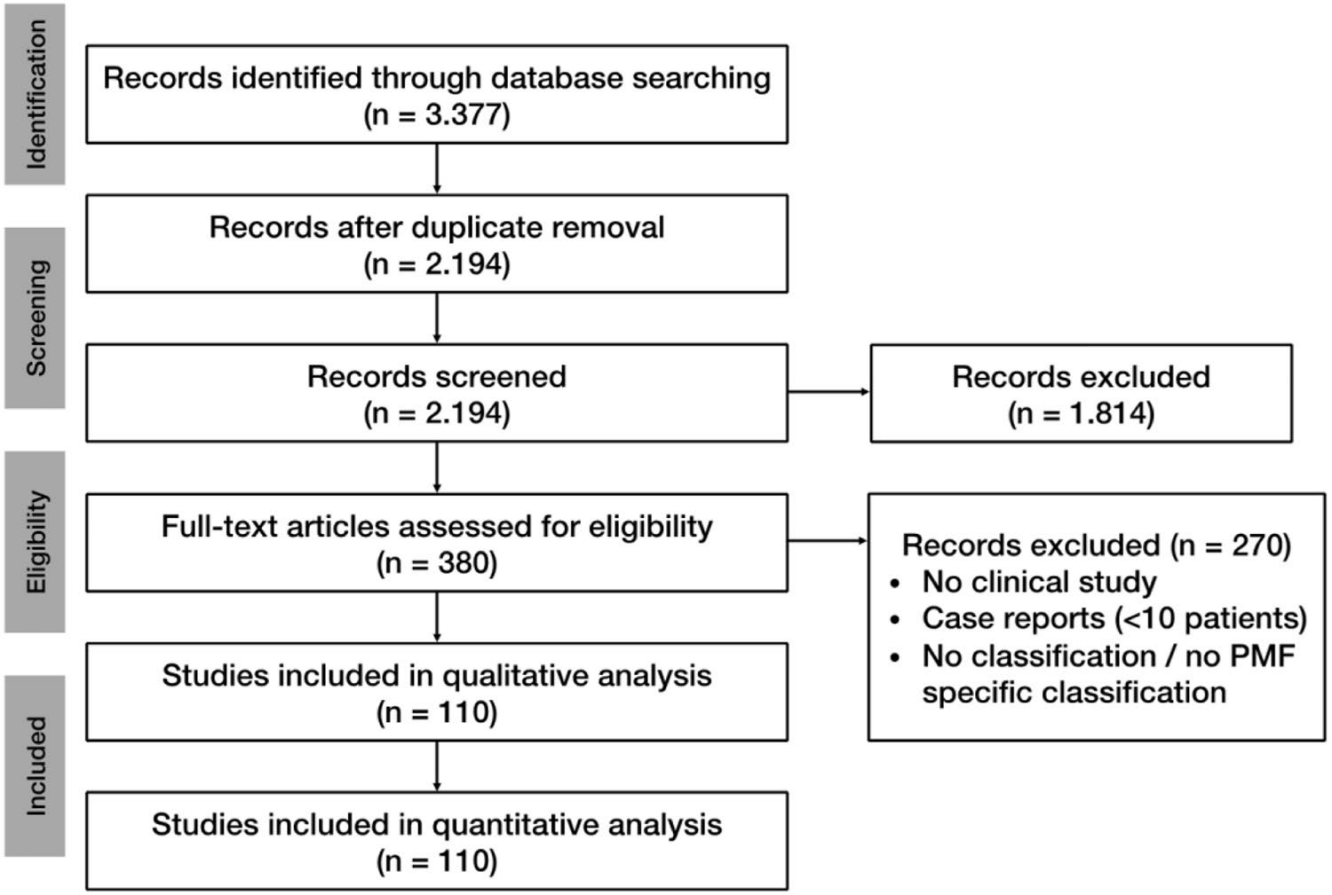
PRISMA flow chart

Evaluation of the databases revealed 3.377 studies potentially relevant for inclusion. After excluding duplicates, title and abstract of the remaining studies were assessed. 380 studies were eligible for full-text analysis, after applying the exclusion criteria (no clinical study, case reports < 10 patients, no classification/no PMF-specific classification), 110 remaining relevant studies were included in this review. The selection process was performed according to “Preferred Reporting Items for Systematic Review and Meta-Analyses” (PRISMA) and is shown in Fig. 1 [33].

### Study characteristics

A total of 110 studies, published between 1965 and 2022, using 143 classification systems, were included. The studies include a number of 12.614 patients with 12.633 ankle fractures and a weighted median age of 44.55 years (13–100). 5.963 patients were female and 5.231 male, 11 studies did not report gender distribution [6, 20, 24, 37–44]. There were 22 prospective studies, 88 retrospective studies; 11 studies were multicenter, and 99 single center studies (Table 1). Detailed information about patient demographics is demonstrated in Table 2. Four specific classifications for the PMF were found: a classification based on the relation of the fragment size compared to the size of the tibial joint surface [45] (referred to as PMF Classification according to fracture size) and 3 CT-based classifications according to Haraguchi, Bartoníček/Rammelt, and Mason [14, 29, 30].

**Table 1:** Overview of the included studies

Study	Study type	Single center vs. multicenter	Modified Coleman methodology score			
	Prospective vs. retrospective		LoE	Part A	Part B	Total
Warner (1965) [37]	RS	SC	IV	17	0	17
McDaniel (1977) [58]	RS	SC	IV	35	7	42
Yde (1980) [99]	RS	SC	IV	15	10	25
Olerud (1986) [38]	RS	SC	IV	38	14	52
Jaskulka (1989) [4]	RS	SC	III	33	15	48
Broos (1991) [8]	PS	MC	IV	23	10	33
Broos (1992) [100]	RS	MC	IV	18	12	30
Heim (2002) [39]	PS	SC	II	22	12	34
Langenhuijsen (2002) [7]	RS	SC	IV	32	21	53
Papachristou (2003) [101]	PS	SC	II	38	17	55
De Vries (2005) [52]	RS	SC	IV	14	21	35
Haraguchi (2006) [14]	PS	SC	IV	12	12	24
Farsetti (2009) [102]	RS	SC	III	34	18	52
Büchler (2009) [13]	RS	SC	IV	5	14	19
Forberger (2009) [103]	RS	SC	IV	34	23	57
Tejwani (2010) [6]	PS	SC	IV	38	23	61
Hong-Chuan (2010) [104]	RS	SC	IV	37	18	55
Heim (2010) [77]	RS	SC	IV	27	21	48
Mingo-Robinet (2011) [46]	RS	SC	II	34	18	52
Purnell (2011) [105]	RS	SC	IV	14	14	28
Abdelgawad (2011) [70]	RS	SC	IV	28	10	38
Xu (2012) [53]	RS	MC	III	20	21	41
Di Giorgio (2013) [106]	RS	SC	IV	30	21	51
Hoelsbrekken (2013) [107]	PS	SC	III	53	26	79
Erdem (2014) [108]	PS	SC	II	47	23	70
Hong (2014) [109]	RS	SC	IV	15	21	36
Bartonicek (2015) [29]	RS	MC	IV	15	19	34
Evers (2015) [47]	RS	SC	III	14	20	34
Drijfhout van Hooff (2015) [71]	RS	SC	IV	23	21	44
Kim (2015) [110]	PS	SC	IV	40	26	66
Verhage (2015) [111]	RS	SC	III	20	23	43
Mangnus (2015) [20]	RS	SC	IV	9	11	20
Choi (2015) [112]	RS	SC	IV	34	16	50
de Muinck Keizer (2016) [40]	RS	SC	IV	8	6	14
Endo (2016) [113]	PS	SC	II	40	20	60
Chan (2016) [114]	RS	SC	IV	25	12	37
Guo (2017) [54]	RS	SC	III	40	19	59
Naumann (2017) [115]	RS	MC	IV	21	21	42
Vidovic (2017) [116]	PS	SC	II	49	23	72
Bali (2017) [64]	PS	MC	IV	35	31	66
Shi (2017) [117]	PS	SC	II	47	28	75
Zhong (2017) [118]	RS	SC	III	34	21	55
Mason (2017) [30]	RS	SC	III	25	24	49
Saygili (2017) [55]	RS	SC	III	32	18	50
Zhou (2017) [119]	RS	SC	IV	30	15	45
Wang (2017) [120]	RS	SC	IV	37	21	58
Xing (2018) [121]	RS	SC	IV	30	12	42
Baek (2018) [122]	PS	SC	II	40	19	59
Levack (2018) [123]	RS	SC	III	40	21	61
Kumar (2018) [80]	PS	SC	IV	19	19	38
Kim (2018) [124]	RS	SC	IV	30	20	50
Yi (2018) [95]	RS	SC	IV	12	14	26
Tosun (2018) [51]	RS	SC	III	27	18	45
Miller (2018) [125]	RS	SC	III	28	12	40
Huang (2018) [126]	RS	SC	III	15	14	29
Zhang (2018) [82]	RS	SC	III	9	14	23
Xing (2018) [127]	RS	SC	III	34	15	49
Sobol (2018) [41]	RS	SC	III	18	12	30
Baumbach (2019) [48]	RS	SC	III	30	14	44
Bartonicek (2019) [128]	RS	MC	IV	9	14	23
Jayatilaka (2019) [43]	PS	MC	IV	19	5	24
Blom (2019) [19]	RS	SC	III	22	21	43
Vosoughi (2019) [67]	RS	SC	III	9	12	21
Hendrickx (2019) [129]	RS	SC	IV	15	14	29
Mason (2019) [68]	RS	SC	IV	32	21	53
Kang (2019) [49]	PS	SC	II	47	26	73
Kellam (2019) [130]	RS	SC	III	12	14	26
Meijer (2019) [131]	PS	SC	II	23	28	51
Sheikh (2019) [60]	RS	SC	IV	5	14	19
Meijer (2019) [132]	RS	SC	III	23	23	46
Testa (2019) [133]	RS	SC	iV	34	21	55
Mitchell (2019) [42]	RS	SC	IV	18	9	27
Verhage (2019) [72]	RS	SC	IIB	28	23	51
Philpott (2020) [44]	RS	SC	IV	30	14	44
Lee (2020) [134]	RS	SC	III	33	21	54
Wang (2020) [61]	RS	SC	III	15	14	29
Pilskog (2020) [56]	RS	SC	III	37	21	58
Zhang (2020) [135]	RS	SC	IV	12	14	26
Blom (2020) [63]	RS	SC	IV	17	23	40
Yang (2020) [136]	PS	SC	IV	28	18	46
Wang (2020) [137]	RS	SC	III	9	12	21
Wang (2020) [138]	RS	SC	III	40	21	61
Sultan (2020) [75]	RS	SC	IV	18	14	32
Lee (2020) [139]	RS	SC	III	12	14	26
Mertens (2020) [65]	PS	SC	IV	48	26	74
Gandham (2020) [83]	RS	SC	IV	15	9	24
McHale (2020) [50]	RS	SC	III	17	23	40
He (2020) [140]	RS	SC	IV	9	14	23
Liu (2020) [141]	PS	SC	II	47	23	70
Palmanovich (2020) [62]	RS	SC	III	17	12	29
Martin (2021) [142]	PS	SC	IV	40	28	68
Yu (2021) [143]	PS	SC	III	44	33	77
Tucek (2021) [66]	RS	SC	IV	30	18	48
Maluta (2021) [1]	RS	SC	IV	30	21	51
Ceccarini (2021) [144]	RS	SC	III	38	21	59
Neumann (2021) [21]	RS	SC	III	35	21	56
Quan (2021) [79]	RS	SC	III	12	12	24
Black (2021) [145]	RS	MC	IV	15	12	27
Fidan (2021) [146]	RS	SC	IV	29	16	45
Erinç (2021) [147]	RS	SC	III	40	19	59
Sun (2021) [98]	RS	SC	IV	26	29	55
Ræder (2021) [59]	RS	MC	III	21	20	41
Xie (2021) [28]	RS	SC	III	25	10	35
Schoenmakers (2021) [78]	RS	SC	III	15	23	38
Seo (2022) [148]	RS	SC	III	15	12	27
Kleinertz (2022) [32]	RS	SC	IV	26	12	14
Yamamoto (2022) [57]	RS	MC	III	30	20	50
Patton (2022) [149]	RS	SC	IV	20	12	32
Pflüger (2022) [24]	RS	SC	IV	18	11	29
Sun (2022) [150]	RS	SC	IV	30	21	51

**Table 2:** Patient demographics

Number of patients	12.614
Median age in years, (range)	44.55 (13–100)
Female, *n* (%)	5.963 (53.2)
Male, *n* (%)	5.231 (46.7)
PMF classification according to fracture size	
Number of patients	8.506
Median age in years, (range)	46.67 (13–100)
Female, *n* (%)	4.035 (52.6)
Male, *n* (%)	3.624 (47.3)
Fracture ≥ 25% of joint surface	1.095
Fractrure < 25% of joint surface	1.579
Other cut-off value used for fracture	1.117
Fracture cut-off value not stated	2.199
Haraguchi classification	
Number of patients	3.790
Median age in years (range)	46.8 (13–100)
Female, *n* (%)	1.699 (52.9)
Male, *n* (%)	1.508 (47.0)
HC Type I	1.525
HC Type II	910
HC Type III	318
Bartonicek/Rammelt classification	
Number of patients	2.497
Median age in years (range)	47.0 (14–90)
Female, *n* (%)	1.282 (55.6)
Male, *n* (%)	1.022 (44.3)
BRC type 1	145
BRC type 2	917
BRC type 3	458
BRC type 4	348
BRC type 5	7
Mason classification	
Number of patients	1.164
Median age in years (range)	48.55 (17–90)
Female, *n* (%)	479 (59.5)
Male, *n* (%)	326 (40.4)
MC type 1	149
MC type 2A	248
MC type 2B	263
MC type 3	124

*HC* Haraguchi classification, *BRC* Bartonicek/Rammelt classification, *MC* Mason classification

### PMF classification according to fracture size

Sixty-six studies that used the size of the PMF in relation to the joint surface as a classification could be included. Of these, 35 studies used radiographs and 30 studies used CT to estimate size, one study did not provide a clear statement in this regard. Only one studied inter- and intraobserver reliability, measuring a substantial Kappa of 0.64 and 0.63 respectively [13]. The majority of these studies used either a cut-off value of 25% for fixation of the PMF (26 studies) or fixed the posterior malleolus regardless of size (28 studies). The remaining studies used either 20% (4 studies), 30% (2 studies), or > 1/3 of the joint area (5 studies) as the cut-off value, 1 study fixed the PMF in young patients or in the presence of subluxation from 10%, and 3 studies did not provide any information (Table 3). Nine studies reported a better outcome with reduction of smaller posterior malleolus fragments [4, 6, 7, 46–51], whereas seven studies reported no difference between fixation and no fixation of smaller posterior malleolus fragments [52–58].

**Table 3:** Studies reporting on the “Size Classification”

Study	Patients/ ankles	Sex/ age	Imaging	Description	PMF type distribution	Treatment allocation	Cut-off	Assesing Classification	Reliability		Predictive outcome values
									Inter OR	Intra OR	
Warner (1965) [37]	n.r./100	n.r	x-ray	Percentage of tibial articular surface	Unclear	0–20%: closed reduction, 20–30% only fix MM/LM, 30–60%: ORIF; via transfibular approach	≥ 20%	No	n.r	n.r	n.r
McDaniel (1977) [58]	50/51	13m, 37f, 41 (20–68) yo	x-ray	Percentage of tibial articular surface	< 10% (9), 10–25% (27), ≥ 25% (15)	Closed methods (28), treated operatively (23; 7 screw fixation PMF ≥ 25%)	≥ 25%	Yes	n.r	n.r	In PMF ≥ 25% ORIF associated with better outcome than closed treatment
Yde (1980) [99]	487/488	263m, 224f, 44 yo	x-ray	Percentage of joint surface	SER3: Only 14% of cases PMF ≥ 25%; PER4: In 73% of cases PMF ≥ 25%	n.r	≥ 25%	No	n.r	n.r	n.r
Olerud (1986) [38]	134/134	Not clear/48 (15–86) yo	x-ray	Percentage of joint surface	86 of 134 with PMF, < 1⁄6 of surface (49), 1⁄6 -1/3(32), ≥ 1/3(5)	Posterior fragments either left alone or fixed with screw	No cut- off	No	n.r	n.r	PMF without displacements best
Jaskulka (1989) [4]	142/142	47m, 95f, 46.7 yo	x-ray	Percentage of articular surface	62 of 142 with PMF: avulsion of posterior Lip (32), PMF 5–20% (15), PMF ≥ 20% (15)	14 ORIF with screws (none of the posterior lip); if < 20% generally no osteosynthesis	No cut- off	Yes	n.r	n.r	Longterm outcome poorer if fracture of posterior margin present (even small fragments); poorest results: conservativly treated PMF
Broos (1991) [8]	604/612	320m, 284f, 41 yo	x-ray	One-third of the joint surface	178 of 612 fractures with PMF	100 of 178 patients with PMF fixed with screws; PMF < 1/3 left untouched	≥ 1/3	Yes	n.r	n.r	If PMF present only 66% excellent or good result compared with 81% without PMF; ankle fx with PMF > 1/3 with perfect anatomic fixation, give worse results than ankle fractures with small unfixed PMF
Broos (1992) [100]	604/612	320m, 284f, 41 yo	x-ray	One-third of the joint surface	178 of 612 fractures with PMF	100 of 178 with PMF fixed with screws; PMF < 1/3 left untouched	≥ 1/3	Yes	n.r	n.r	In spite of perfect anatomic internal fixation of fragments > 1/3, the results remained worse than with small non fixed posterior fragments
Heim (2002) [39]	n.r./111	n.r	x-ray	One-third of the articular surface	n.r	Surgically fixed if PMF ≥ 1/3 size	≥ 1/3	No	n.r	n.r	n.r
Langenhuijsen (2002) [7]	57/57	21m, 36f, 49.6 (18–89) yo	x-ray	Percentage of articular surface	PMF < 10% (24); 10–25% (19), > 25% (14)	53 of 57 treated operatively, 4 conservatively; ORIF (14) (1x < 10%, 3 × 10–25%, 10x ≥ 25%)	No cut- off	Yes	n.r	n.r	Size or fixation of fragment did not influence prognosis; joint congruity was significant factor for prognosis, congruity should be achieved for fragments > 10% of the tibial articular surface
Papachristou (2003) [101]	15/15	4m, 11f, 48.8 (14–73) yo	x-ray	Perecntage of articular surface	All between 1/4—1/3 of articular surface	All ORIF PL or PM approach	≥ 25%	No	n.r	n.r	n.r
De Vries (2005) [52]	45/45	10m, 35f, 61 (37–81) yo	x-ray	Percentage of articular surface	PMF < 25% (32), PMF > 25% (13)	11 of 45 patients with PMF fixation: PMF ≥ 25% (8), PMF < 25% (3)	≥ 25%	Yes	n.r	n.r	PMF fixation did not have better outcome than not fixated (AFSS); no evidence for the need for fixation of fragments smaller than 25%
Haraguchi (2006) [14]	57/57	41m, 16f, 43 (13–80) yo	CT	Percentage of tibial plafond	9 of 57 Fx ≥ 25% (7 of 9 extended to medial malleolus)	All fractures treated with ORIF, best approached medially because of medial extension	≥ 25%	No	n.r	n.r	n.r
Farsetti (2009) [102]	44/44	19m, 25f, 43 (18–69) yo	x-ray	Percentage of joint surface length	26 trimalleolar fractures	ORIF if PMF > 25% (10)	≥ 25%	No	n.r	n.r	n.r
Büchler (2009) [13]	22/22	7m, 15f, 50 (21.9–72.2) yo	x-ray + CT	Percentage of articular surface	All trimalleolar fractures, 19 ankles (86%) with a PM lesion of tibial plafond	n.r	No cut- off	Yes	Kap-pa 0.64	Kappa 0.63	n.r
Forberger (2009) [103]	45/45	12m, 33f, 54 (18–83) yo	x-ray	Percentage of articular surface	PMF ≥ 25% (19); PMF ≥ 10% surgically treated if younger (under 50yo) or with subluxation (n = 26)	PL approach used for osteosynthesis in 45 patients	≥ 25% ≥ 10% if sub-luxation or < 50 yo	No	n.r	n.r	n.r
Tejwani (2010) [6]	309/n.r	n.r./43.3 yo	x-ray	Percentage of fragment size	54 of with PMF	Fixation decision by surgeon based on size and tibio-talar stability: fixation (20) average size 25.2%; not fixed (34) average size 16.1%	No cut- off	No	n.r	n.r	Trend for better outcome scores for patients who had fixation (numbers too small to meaningfully compare fixation versus no fixation groups)
Hong-Chuan (2010) [104]	96/96	75m, 21f, 38 (17–79) yo	x-ray	Percentage of articular surface	PMF < 25% (10); PMF ≥ 25% (26)	PMF ≥ 25 fixed (4 × PM approach, 3 × PL approach, 19 × medial approach; PMF < 25% reduced spontane. after reduction of LM	≥ 25%	No	n.r	n.r	n.r
Heim (2010) [77]	43/43	15m, 28f, 58.8 yo	x-ray	Percentage of articular surface	No PMF (7), small shelllike (10), PMF < 25% (18), PMF ≥ 25% (8)	8 PMF ≥ 25% fixed: AP screw (4), PA screw (1), plate (3)	No cut- off	Yes	n.r	n.r	Volkmann fx worse prognosis; fixation with anatomic results does not return score to norm (OMAS), instability as indicator for PMF fixation
Mingo-Robinet (2011) [46]	45/45	10m, 35f, 50.69 ± 17.32 (20–80) yo	x-ray	Percentage of articular surface	PMF < 25% (20), PMF ≥ 25% (25)	Ligamentotaxis (27), AP fixation (15), PL fixation (3)	No cut- off	Yes	n.r	n.r	Tendency toward worse result when PMF ≥ 25%; better radiological results when small PMF reduced; results encourage to fix all PMF ≥ 25% and fix most of smaller PMF not satisfactorily reduced by ligamentotaxis
Purnell (2011) [105]	66/67	45m, 21f, 44 (18–69) yo	CT	Percentage of articular surface	23 of 67 with PMF	PMF fixed if ≥ 25% (AP screws after closed reduction)	≥ 25%	No	n.r	n.r	n.r
Abdelgawad (2011) [70]	12/12	10m, 2f, 41 (20–61) yo	x-ray	Percentage of tibial plafond	All ≥ 30% of tibial plafond	Fixation of PMF done if ≥ 30% of tibial plafond and more than 2 mm displaced after closed reduction (12) with PL approach	≥ 30%	No	n.r	n.r	n.r
Xu (2012) [53]	102/102	41m, 61f, 43.4 (15–80) yo	x-ray	Percentage of articular surface	PMF < 10% (16), PMF 10–25% (62), PMF ≥ 25% (24)	42 of 102 surgically fixed; PMF < 25% fixed (18) unfixed (60)	No cut- off	Yes	n.r	n.r	Arthritis score for different PMF sizes differed greatly; difference in treatment effect between articular surface evenness and unevenness for all fragment; no difference in treatment effect between fixed and unfixed PMF (AOFAS; VAS)
Di Giorgio (2013) [106]	18/18	12m, 6f, 44.2 (26–61) yo	x-ray	Percentage of articular surface	8 of 18 with PMF; ≥ 25% (8)	Fixating PMF ≥ 25% and ignoring smaller fragments followed in 82% of patients (21); in 5 of 13 (38%) PMF ≥ 25% not fixated	≥ 25%	No	n.r	n.r	n.r
Hoelsbrekken (2013) [107]	82/82	31m, 51f, 52.8 (± 15,3) yo	x-ray	Percentage of articular surface	n.r	ORIF of PMF if the fragment ≥ 25% of the articular surface	≥ 25%	No	n.r	n.r	n.r
Hong (2014) [109]	31/31	10m, 21f, 46 (20–76) yo	x-ray	Percentage of articular surface	PMF < 25% (21), PMF ≥ 25% (10); mean size 22.3% (range 6% to 46%)	8 of 31 PMF fixed (≥ 25%)	≥ 25%	No	n.r	n.r	Poorer functional outcome with increasing posterior malleolar fragment size (OMAS score)
Erdem (2014) [108]	40/40	20m, 20f, 48.9 (24–51) yo	x-ray + CT	Percentage of articular surface	n.r	Surgical treatment if fracture > 20%, screws (20), plate (20)	≥ 20%	No	n.r	n.r	n.r
Evers (2015) [47]	42/42	16m, 26f, 52.8 (19–86) yo	x-ray + CT	Percentage of joint surface involvement	Group I: 54,8% < 25% (23), group II: 45% ≥ 25% (19)	< 25% osteosynthesis (2), ≥ 25% osteosynthesis (14)	No cut- off	Yes	n.r	n.r	Patients with PMF fixation had higher probability achieving good AOFAS Score and therefore improved outcomes
Kim (2015) [110]	36/36	16m, 20f, 58 (23–85) yo	x-ray	Percentage of articular surface	All ≥ 30% of the tibial plafond	All lateral transmalleolar approach and miniscrews fixation	≥ 30%	No	n.r	n.r	n.r
Verhage (2015) [111]	243/243	114m, 129f, 52 yo	x-ray	Percentage of articular surface	Mean PMF size 16% (range 3–53%)	Fixation in 11 cases: 8x ≥ 25%, 3 × 5–25%, large posterior fragments reduced closed or percutaneously and fixed by 1- 2 AP screws	No cut- off	Yes	n.r	n.r	Fragments > 5% resulted in worse functional outcome than fragments < 5% (not statistically significant)
Choi (2015) [112]	50/50	27m, 23f, 47.5 (25–75) yo	CT	Percentage of articular surface	Mean area 31.7%	ORIF with single oblique PL approach; included involvement of PMF < 25%	No cut- off	No	n.r	n.r	n.r
Drijfhout van Hooff (2015) [71]	131/131	55m, 76f, 51 (24–74) yo	x-ray	Percentage of joint surface	Small (< 5%, *n* = 20), medium (5–25%, *n* = 86), or large (≥ 25%, *n* = 25)	In 24 patients PMF fixed (mainly percutaneously in AP direction)	No cut- off	Yes	n.r	n.r	No difference in outcome comparing small PMF to medium and large fragments (AOFAS, AAOS, VAS,RoM); patients with PMF > 5% developed more OA; anatomical fixation of PMF between 5 and 25% may lead to improved outcome
Endo (2016) [113]	20/20	15m, 5f, 47 (18–67) yo	CT	Percentage of articular surface	n.r	PMF ≥ 25% fixed with 1—2 screws; PMF < 25% of the articular surfaces were fixed depending on surgeons preference	No cut- off	No	n.r	n.r	n.r
Chan (2016) [114]	254/254	106m, 148f, 45.1 (13–83) yo	x-ray + CT	One-third of the articular surface	95 of 256 with PMF	Only 13 with fixation (fixed with guide pins through PL approach if displaced > 2 mm and involved > 1/3 size)	≥ 1/3	No	n.r	n.r	n.r
Guo (2017) [54]	284/284	156m, 128f, 39.12 (± 12.23) yo	CT	Percentage of joint surface involve-ment	< 25% (239), ≥ 25% (44)	Fixation ≥ 25% (40) < 25% (72) AP screws; no fixation ≥ 25% (4) < 25% (168) conservatively	No cut- off	Yes	n.r	n.r	PMF < 25%: no difference in outcomes fixed vs. not fixed in tibial spiral fx; PMF > 25% significantly improved clinical outcome with fixation (AOFAS; VAS)
Naumann (2017) [115]	1011/1011	455m, 556f, 51.4 (18–94) yo	x-ray	≥ ¼ of the articular surface	235 of 1011 with PMF	ORIF if PMF ≥ 1/4 of the articular surface	≥ 25%	No	n.r	n.r	n.r
Vidovic (2017) [116]	46/46	28m, 18f, 52.3 (33–71) yo	CT	Percentage of articular surface	All ≥ 25% of articular surface	Indirect reduction and percutaneous AP fixation (22); direct reduction and PA fixation (24)	≥ 25%	No	n.r	n.r	Significantly better quality of reduction with direct reduction
Shi (2017) [117]	116	59m, 57f, 48.6 (± 13.36) yo	x-ray + CT	Percentage of articular surface	All ≥ 25% of articular surface	Direct fixation (plate or screw) (64), indirect fixation (ligamentotaxis) (52)	≥ 25%	No	n.r	n.r	Direct reduction technique provide better quality of fracture reduction and outcome of PMF > 25% (AOFAS)
Saygili (2017) [55]	73/73	34m, 39f, 42.65 (± 13.68) yo	x-ray	Percentage of joint surface	All < 25% of joint surface	PMF fixation (10 × screw, 17 × plate), no fixation (46)	≥ 25%	Yes	n.r	n.r	PMF fixation vs no fixation had no significant difference (AOFAS)
Zhou (2017) [119]	34/34	15m, 19f, 41.2 (21–71) yo	3DCT	Percentage of distal articular tibia	All ≥ 25% of articular surface (1/4–1/3(19), 1/3–1/2 (11), ≥ 1/2 (4))	ORIF: plates and screws via a PL approach	≥ 25%	No	n.r	n.r	n.r
Wang (2017) [120]	84/84	39m, 45f, 44.16 (15–75) yo	CT	Percentage of articular surface	40 of 84 patients with PMF ≥ 25%	PMF ≥ 25% fixed with 2 screws via PL approach; PMF remain untreated when fracture < 25%	≥ 25%	No	n.r	n.r	n.r
Xing (2018) [121]	30/30	19m, 11f, 30 (22–65) yo	x-ray + CT	Percentage of articular surface	All ≥ 25% of articular surface	PMF fixed with screw or Kirschner wire	≥ 25%	No	n.r	n.r	n.r
Baek (2018) [122]	29/29	20m, 9f, 31.3 (17–55) yo	x-ray + CT	Percentage of articular involve-ment	19 of 29 with PMF	n.r	≥ 20%	No	n.r	n.r	71.4% of the patients with malreduced syndesmosis had associated PMF without fixation since < 20% size
Levack (2018) [123]	178/178	63m, 115f, 50.2 (16.9) yo	CT	Percentage of 2-D tibial plafond area	122 of 178 with PMF: 10% (60%), 10%–20% (28%), ≥ 20% (12%)	PMF reduced and with plate regardless of fracture size	No cut- off	No	n.r	n.r	n.r
Kim (2018) [124]	19/19	7m, 12f, 45.1 (13–68) yo	x-ray	Percentage of articular surface	All ≥ 25% of articular surface	Release of PITFL through PL approach for PMF reduction with posterior screws (19)	≥ 25%	No	n.r	n.r	n.r
Tosun (2018) [51]	49/49	21m, 28f, 47 (20–82) yo	CT	Percentage of articular surface	Mean size of PMF: 21.3% in group 1, 28.9% in group 2	29 × untreated PMF; group 2: PMF treated regardless size (surgeons preference) 8 of 20 PMF < 25% fixation: screw or plate	No cut- off	Yes	n.r	n.r	Recommend all PMFs to be fixed regardless of size (related to successful radiological and functional outcomes, AOSAF, OA)
Miller (2018) [125]	198/198	69m, 129f, 48.86 (± 15,98) yo	x-ray	Percentage of articular width	Mean in prone group (n = 47): 22.2%, mean in supine group (n = 151): 15.2%	Prone: 100% fixation (46 × plate, 1 × syndesmotic); supine: 24.5% fixation (37) (2 × plate, 41 × syndesmotic)	No cut- off	No	n.r	n.r	n.r
Huang (2018) [126]	111/111	72m,39f, 46.6 ± 16.1 yo	CT	Percentage of articular surface	42 of 111 with PMF	Surgical treatment (19) PMF ≥ 25%, conservative treatment (23)	≥ 25%	No	n.r	n.r	n.r
Xing (2018) [127]	69/69	41m,28f, 37.36 (20–67) yo	x-ray + CT	Percentage of articular surface	All at least 25% of the articular surface	PMF anatomically reduced by screw	≥ 25%	no	n.r	n.r	n.r
Baumbach (2019) [48]	236/236	99m, 137f, 53.0 (± 18.3) (18–100) yo	CT	Percent tibial plafond depth	< 25% (169), ≥ 25% (67)	ORIF (48 < 25%, 30 > 25%), CRIF (14 < 25%, 30 > 25%), none (107 < 25%, 7 > 25%)	No cut- off	Yes	n.r	n.r	All PMF independent of size should be treated by ORIF, as this restores syndesmotic stability significantly more often than untreated PMF
Kang (2019) [49]	62/62	35m, 27f, 50.7 (19–78) yo	CT	Percentage of tibial joint surface	All affecting > 10%—< 25% of the tibial joint surface	32 × screw fixation; 30 × internal fixation malleolar fractures and no screw fixation PMF	No cut- off	Yes	n.r	n.r	Surgical fixation of PMF < 25% results in better clinical outcome (AAOS, FS-36, FAOS)
Meijer (2019) [132]	104/104	41m, 63f, 63 (37–91) yo	x-ray	Percentage of articular surface	n.r	25% cut-off; fixation of PMF in 19 fixated (18%) with AP or PA screw	≥ 25%	No	n.r	n.r	n.r
Meijer (2019) [131]	31/31	12m, 19f, 46 (19–73) yo	Q3DCT	Percentage of articular surface	Mean articular surface of 12% (range 0.0%–36.0%) of the joint surface	All ORIF: direct fixation (14), syndesmotic screw (4), no fixation (13)	No cut- off	Yes	n.r	n.r	Fragment size was correlated with arthrosis; no significant correlations were found for FAOS and SF-36 scores
Testa (2019) [133]	48/48	22m, 26f, 44.7 (19–72) yo	x-ray	Percentage of articular surface	Mean fragment size 18%, mean fragment size fixing 26%, 13 of 48 ≥ 25%	Fixation of PMF in 8 of 48 when PMF ≥ 25%, fixed with screws	≥ 25%	No	n.r	n.r	No correlation between size of PMF and ankle dislocation and size of PMF and syndesmosis stability
Verhage (2019) [72]	169/169	67m, 102f, 52.3 yo	x-ray	Percentage of intra-articular surface	< 5% (20), 5–25% (119), ≥ 25% (30)	39 × Fixation of PMF: none of < 5% were fixated, 15 of the 5–25% fixated,24 of ≥ 25% fixated	No cut- off	Yes	n.r	n.r	Size and fixation of PMF not associated with OA or outcome; postoperative step-off as risk factor
Pilskog (2020) [56]	130/130	36 m, 94f, 57 (41–67) yo	x-ray	Percentage of joint involve-ment	Median PMF size 17%; 65 PMF < 25% (31 × fixation, 35 × no fixation)	PMF fixation in 42 of 43 (98%) in group A, 7 of 43 (16%) in group B	No cut- off	Yes	n.r	n.r	PMF < 25% fixed vs. not-fixed same SEFAS, RANS-36, VAS
Blom (2020) [63]	70/70	30m, 40f, 47 (SD 14.6) yo	CT	Percentage of tibial articular surface	Mean of 15.9% of articular surface	According to AO principles of ORIF	No cut- off	Yes	n.r	n.r	PMF morphology and not size found as predictor of FAOS score
Wang (2020) [138]	243/243	120m, 123f, 44.27 (± 12,5) yo	CT	FAR from CT crossectional images	≥ 15% (136) and < 15% (107)	All ORIF: reduction of PMF via PL approach, PA/ AP screws and PA plate used	No cut- off	No	n.r	n.r	n.r
McHale (2020) [50]	75/75	24m, 51f, 56.2 yo	x-ray	Percentage of fragment size	< 10% (34), 10–20% (18), 20–30% (12), ≥ 30% (11)	25 of 75 fractures fixed (surgeons preference) using PL approach (22 × plate, 3 × PA screw)	No cut- off	Yes	n.r	n.r	Worse outcome (MOXFQ, EQ-5D VAS) when PM size 10–20%, due to higher rate of anatomic reduction when fixed; best PROMs in PMF < 10%
Lee (2020) [134]	166/166	97m, 69f, 44.6 (18–83) yo	CT	Percentage of intra-articular surface	92 of 166 Fx with PMF (51 ≥ 25%)	Screw fixation PMF ≥ 25% and no long proximal extension; additional plate with PMF ≥ 25% and long proximal extension	≥ 25%	No	n.r	n.r	Incidence of chronic syndesmotic instability higher, regardless of PMF fixation
Liu (2020) [141]	85/85	48m, 37f, 42.92 (21–66) yo	not clear	Percentage of articular surface	All ≥ 25%; exclusion: medial malleolus extension type of PMF	plate group (n = 47), screw group (n = 38)	≥ 25%	No	n.r	n.r	n.r
Palmanovich (2020) [62]	85/85	23m,62f, 53.4 (17–89) yo	CT	1/3 of antero-posterior curvature of articular surface	All over 1/3 articular surface	Single PA screw fixation	≥ 1/3	No	n.r	n.r	n.r
Lee (2020) [139]	70/70	41m, 29f, 51.0 (18–78) yo	CT	Percentage of articular surface	n.r	PMF not fixed if no rotation or displacement, if PMF ≥ 20% fixation	≥ 20%	No	n.r	n.r	n.r
Ceccarini (2021) [144]	135/135	82m, 53f, 38.2 (19–50) yo (9 lost to FU)	x-ray	Percentage of articular surface	n.r	≥ 25% fixation with screw in AP or PA direction	≥ 25%	No	n.r	n.r	n.r
Schoen- makers (2022) [78]	26/26	10m, 16f, 58.08 ± 18.9 (22–78) yo	CT	Percentage of surface area	< 10% (6), 10%–25% (14), > 25% (6)	Surgical treatment 14 of 26 (9 × 10–25%, 4x > 25%)	No cut- off	Yes	n.r	n.r	Optimal value for PMF size to predict excellent AOFAS score was < 15%; PMF size ≤ 10% had a significantly greater RoM
Yamamoto (2022) [57]	110/110	44m, 66f, 52.5 ± 12.9 yo	CT	Percentage of intra-articular surface	< 25% ( 62), > 25% (48)	n.r	No cut- off	Yes	n.r	n.r	PMF < 25% fragment reduction, fixation, and ankle stability not associated with AOFAS score;
Pflüger (2022) [24]	193/193	n.r	CT	Proportion of PMF in relation to tibial diameter	0%-10% (11), 11%-15% (20), 16%-20% (30), 21%-25% (54), 26%-30% (36), 31%-35% (15), > 35% (27)	Surgical fixation PMF with larger size compared to without, no influence of size on direct or indirect fixation method	No cut- off	Yes	n.r	n.r	n.r

### Haraguchi classification

**Fig. 2:**
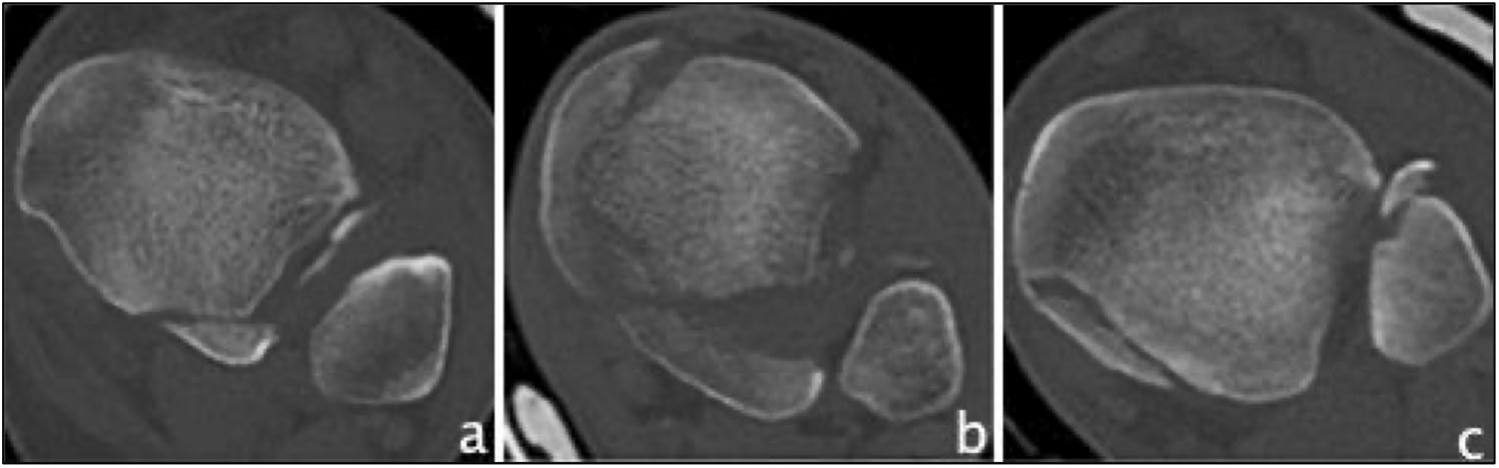
Overview of the Haraguchi classification based on CT images (axial views). **a** Haraguchi type I **b** Haraguchi type II **c** Haraguchi type III

The first CT-based classification found, was developed 2006 by Haraguchi et al*.* which classified PMF into 3 distinct types [14]. Type I is described as a posterolateral-oblique wedge-shaped fragment involving the posterolateral corner of the tibial plafond, type II as a transverse medial-extension fracture line extending from the fibular notch to the medial malleolus, and type III is characterized as a small-shell type fragment at the posterior lip of the tibial plafond (Fig. 2). So far, Haraguchi's classification has been mentioned in 101 studies and was applied in 44 of them, which were, therefore, included and can be seen in Table 4. Three studies reported on the reliability of the classification, all showing substantial interobserver reliability (Fleiss kappa 0.70/Cohen’s kappa 0.799/Cohen’s kappa 0.797) and substantial to almost perfect intraobserver reliability (Fleiss kappa 0.77/Cohen’s kappa 0.985) [24, 32, 59]. Modifications of the Haraguchi classification were found three times. Kumar et al*.* divided Haraguchi type II into subtype A: a single fracture line extending from the fibular notch of the tibia to the medial malleolus, and subtype B: a posterior fracture lines forming 2 separate fragments, which was also applied by Sheikh et al*.* [60]. Wang et al*.* also modified Haraguchi type II by categorizing the fracture line into an anterolateral oblique line (subtype I) and into a small avulsion (subtype II) [61]. Palmanovich et al*.* divided the posterior segment by a central line, perpendicular to the bimalleolar line, into medial and lateral sub-segments, creating a 4-quadrant grid; each posterior malleolar fracture was then categorized based on the fragment’s location into “postero-lateral”, “postero-medial” and “postero-central” [62]. In terms of predictive values, type II fractures were regarded to show worse outcome [19, 59, 63], have higher presence of osteoarthritis [59], and are more likely to require placement of 2 syndesmotic screws [41]. The use of a posteromedial approach for type II fractures have resulted in good Olerud and Molander ankle score (OMAS)[64]. Mertens et al*.* observed an improving AOFAS score from type I to type III [65], Xie et al*.* found most intercalary fragments (more than 2/3) in type I fractures [28], and Kang et al*.* reported a better outcome with surgical treatment of type I fractures smaller than 25% [49].

**Table 4:** Studies reporting on the Haraguchi classification

Study	Patients/ ankles	Sex/ age	PMF type distribution	Treatment allocation	Assesing Classification	Reliability		Predictive outcome values
						Inter OR	Intra OR	
Haraguchi (2006) [14]	57/57	41m, 16f, 43 (13–80) yo	Type I (38), type II (11), type III (8)	Type I: ORIF only when persistent intra-articular displacement; type II: only fixation of medial fragment and one-part fragments	Yes	n.r	n.r	n.r
Erdem (2014) [108]	40/40	20 m, 20f, 48.9 (24–51) yo	Type I (28), type II 10), type III (2)	Screws (20), plate (20) via PL approach	No	n.r	n.r	n.r
Mangnus (2015) [20]	45/45	n.r	Type I (13), type II (15), type III (17)	n.r	Yes	n.r	n.r	n.r
Choi (2015) [112]	50/50	27m, 23f, 47.5 (25–75) yo	Type I (21), type II (29), type III (0)	Single oblique PL approach for ORIF (37 × screw, 3 × buttress plate)	No	n.r	n.r	n.r
de Muinck Keizer (2016) [40]	28/28	n.r	Type I (17), type II (8), type III (3)	n.r	No	n.r	n.r	n.r
Bali (2017) [64]	15/15	9m, 6f, 37.8 yo	Only type II	PM approach for type II fixation	No	n.r	n.r	Good functional outcome using posteromedial approach (OMAS)
Zhong (2017) [118]	48/48	18m, 30f, 43.4 ± 12.7 (18–64) yo	Type I (26), type II (13), type III (9)	AP screws using PM approach (20), PA screws using PL approach (28)	No	n.r	n.r	n.r
Saygili (2017) [55]	73/73	34m, 39f, 42.65 ± 13.68 yo	Type I (39), type II (28), type III (6)	ORIF: posterior plate (17) percutaneous screw fixation (10)	Yes	n.r	n.r	Correlation was found between PMF ratio and Haraguchi type I and II fractures
Yi (2018) [95]	107/107	43m, 64f, 46.58 ± 15.5 yo	Type I (76), type II (30), type III (1)	n.r	No	n.r	n.r	n.r
Kumar (2018) [80]	56/56	48m, 8f, 34.7 ± 11.7 yo	16 of 56 with PMF; type I (9), modified type II into subtypes:A (3);B (1); type III (3)	Prefer a PL incision in type I and a PM incision in type II, both PM and PL incisions preferred in type IIB	Yes	n.r	n.r	n.r
Huang (2018) [126]	111/111	72m,39f, 46.6 ± 16.1 yo	42 of 111 with PMF: type I (41), type II (0), type III (1)	Surgical treatment (19), conservative treatment (23)	No	n.r	n.r	n.r
Kim (2018) [124]	19/19	7m, 12f, 45.1 (13–68) yo	Type I (13), type II (6), type III (0)	Release of PITFL through PL approach for PMF reduction with posterior screws (19)	No	n.r	n.r	n.r
Sobol (2018) [41]	n.r./193	n.r	24 of 193 with PMF: type I (18), type II (6), type III (0)	All type II with 2 AP screws, type I: screws (16), plate (1), no fixation (1); single syndesmotic screw (3 type I, 1 type II)	Yes	n.r	n.r	Type II were significantly more likely than type I fractures to have 2 screws placed
Hendrickx (2019) [129]	164/164	118m, 46f, 41.7 (14–90) yo	36 of 164 patients with PMF, 2 CTs insufficient: type I (33), type II (1), type III (0)	n.r	No	n.r	n.r	Type I was the pattern specific to PMF associated with tibial shaft fractures
Baumbach (2019) [48]	236/236	99m, 137f, 53.0 ± 18.3 (18–100) yo	Type I (112), type II (81), type III (43)	ORIF: type I (44), type II (34), type III (0); CRIF: type I (15), type II (28), type III (1); no fixation type I (53) type II (19), type III (42)	No	n.r	n.r	n.r
Blom (2019) [19]	73/73	30m, 43f, 48 ± 14.8 yo	Type I (20), type II (21), type III (32)	Type I: direct fixation (8), non-direct fixation (6), no fixation (6); type II: direct fixation (13), non-direct fixation (1), no fixation (7); type III: direct fixation (1), non-direct fixation (20), no fixation (11)	Yes	n.r	n.r	Type II: worse outcome (FAOS, symptoms, pain, ADL)
Kang (2019) [49]	62/62	35m, 27f, 50.7 (19–78) yo	Only type I involving < 25% of the articular surface	32 × screw fixation for PMFs (group A), 30 × internal fixation for malleolar fractures without screw fixation for PMFs (group B)	Yes	n.r	n.r	Surgical fixation of type I PMFs involving < 25% of the articular surface results in better clinical outcome (AAOS, FS-36, FAOS)
Kellam (2019) [130]	115/115	52m, 63f, 47 (16–93) yo	Type I (62), type II (43), type III (10)	PMF in tibial shaft fractures: 50 (93%) underwent direct surgical fixation, PMF in ankle fx: 38 (63%) direct surgical fixation	Yes	n.r	n.r	PMF in tibial shaft fx: 14 (47%) of type II injuries had additional fracture line oriented in the sagittal plane (fx pattern unique to these injuries) and more frequently extended to include MM
Meijer (2019) [131]	31/31	12m, 19f, 46 (19–73) yo	Type I (17), type II (7), type III (7)	Type I: fixation (8), no fixation (9); type II both fragments fixed (3)(2xplate, 1 × AP screws),only PL fragment (4); type III: syndesmotic screw (2), no fixation(5)	No	n.r	n.r	n.r
Mitchell (2019) [42]	n.r./122	n.r	59 of 122 with PMF; 44 of 59 with CT data: type I (41), type II (3), type III (0)	30 treated with 1—2 screws, 29 treated nonoperatively	No	n.r	n.r	n.r
Sheikh (2019) [60]	20/20	11m, 9f, 50.4 (22–76) yo	Modified after Kumar: Type I (10), type IIA (5), type IIB (4), type III (1)	n.r	No	n.r	n.r	n.r
Yang (2020) [136]	27/27	11m, 16f, 61.5 (53–67) yo	Only type II	ORIF (combined posterolateral and posteromedial approach)	No	n.r	n.r	n.r
Mertens (2020) [65]	50/50	24m, 26f, 54 (21–83) yo	Only 46 of 50 patients classified: type I (23), type II (20), type III (3)	All ORIF (plate osteosynthesis)	Yes	n.r	n.r	Improving clinical outcomes (AOFAS score) from type I to type III
Blom (2020) [63]	70/70	30m, 40f, 47 ± 14.6 yo	Type I (23), type II (22), type III (25)	Type I: direct fixation (10), non-direct fixation (7), no fixation (6); type II: direct fixation (16), non-direct fixation (1), no fixation (5); type III: direct fixation (1), non-direct fixation (20), no fixation (4)	Yes	n.r	n.r	Type II with poorer FAOS; Type I: quality of reconstruction of tibial articular surface as predictive factor; Type III: quality of syndesmosis stabilization as predictive factor
Wang (2020) [138]	78/78	40m, 38f, 49.05 ± 15.97 yo	Created subtypes of type II: Type I (40), type II (38)	n.r	No	n.r	n.r	n.r
Zhang (2020) [135]	106/106	59m, 47f, 47.3 (21–75) yo	Only type I	n.r	No	n.r	n.r	n.r
Wang (2020) [137, 140]	48/48	13m, 35f, 48.9 (16–82) yo	Type I (25), type II (18), type III (5)	All were treated surgically (33 underwent ORIF: 5 × plates, 28 × screws)	No	n.r	n.r	n.r
He (2020) [140]	41/41	32m, 9f, 37.9 (18–61) yo	34 of 41 patients with PMF: type I (20), type II (4), type III (10)	n.r	No	n.r	n.r	n.r
Palmanovich (2020) [62]	85/85	23m,62f, 53.4 (17–89) yo	Modified Haraguchi: 61 cases “postero-lateral” 4 cases “postero-medial” 20 cases “postero-central”	Mean trajectory angle for single PA screw was 21° lateral for “postero-lateral” fragments, 7° lateral for “postero-central” fragments, and 28° medial for “postero-medial”	Yes	n.r	n.r	n.r
Martin (2021) [142]	28/28	16m, 12f, 36 (19–69) yo	Type I (13), type II (14), type III (1)	Posterior arthroscopic reduction and internal fixation	No	n.r	n.r	n.r
Yu (2021) [143]	76/76	49m, 27f, 47.2 ± 13,5 yo	Only type I	Percutaneous PA and AP screw fixation	No	n.r	n.r	n.r
Quan (2021) [79]	95/95	28m, 67f, 50.07 (20–83) yo	Type I (66), type II (19), type III (10)	n.r	Yes	n.r	n.r	Most multifragment fractures cannot be defined with Haraguchi classification
Black (2021) [145]	279/279	93m, 186f, 55 ± 19 yo	Type I (161), type II (94), type III (24)	n.r	No	n.r	n.r	n.r
Fidan (2021) [146]	65/65	29m, 36f, 39.6 (18–89) yo	Type I (45), type II (12), type III (8)	Posterior plating via PL approach	No	n.r	n.r	n.r
Erinç (2021) [147]	86/86	53m, 33f, 41.70 ± 14.24 yo	Type I (27), type II (59), type III (0)	AP screw (50), ORIF (36)	No	n.r	n.r	n.r
Sun (2021) [98]	46/46	20m, 26f, 52.9 ± 14.1 yo	Type I (11), type II (38), type III (0)	Single-fragment type: posterior plate fixation with PL approach; double-fragment type: AP screw for PL fragment, PA screw for PM fragment	No	n.r	n.r	
Ræder (2021) [59]	210/210	130m, 80f, 44.74 ± 14.63 yo	125 of 210 with PMF: type I (61), type II (28), type III (36)	Syndesmotic stabilization in all patients; 13% treated surgically (5 × type 1, 12 × type 2)	Yes	C.kappa 0.797	n.r	Presence of OA after 2 years ranged from 34% in type III group to 64% in type II group; type II had lower AOFAS score compared to no PMF
Schoenmakers (2022) [78]	26/26	10m, 16f, 58.08 ± 18.9 (22–78) yo	Type I (11), type II (11), type III (4)	Surgical treatment in 14 of 26	Yes	n.r	n.r	Classification showed no relationship with functional outcome
Kleinertz (2022) [32]	113/113	37m, 76f, 56.2 ± 17.8 yo	n.r	n.r	Yes	C.kappa 0.799	C.kappa 0.985	n.r
Yamamoto (2022) [57]	110/110	44m, 66f, 52.5 ± 12.9 yo	Only Type I if > 5% of joint surface and type II; type I (70), type II (40)	n.r	Yes	n.r	n.r	Postoperative complications associated with low AOSAF score but not reduction and fixation
Patton (2022) [149]	153/153	46m, 107f, 51 ± 8 (18–89) yo	Type I (50), type II (49), type III (37), unclassifiable (5)	n.r	No	n.r	n.r	No association to Lauge-Hansen injury mechanism
Pflüger (2022) [24]	193/193	n.r	n.r	n.r	Yes	F.kappa 0.70	F.kappa 0.77	n.r
Sun (2022) [150]	32/32	11m, 21f, 45.6 ± 6.3 (32–59) yo	Type I (20), type II (12), type III (0)	PL approach with distal locking plate	No	n.r	n.r	n.r

### Bartoníček/Rammelt classification

**Fig. 3:**
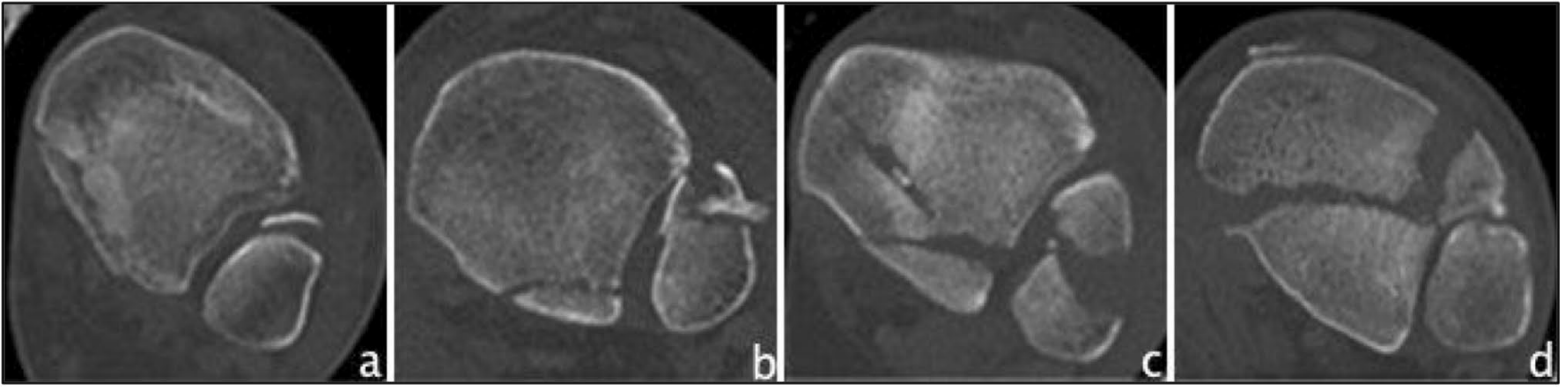
Overview of the Bartoníček/Rammelt classification based on CT images (axial views). **a** Bartoníček type 1 **b** Bartoníček type 2 **c** Bartoníček type 3 **d** Bartoníček type 4

Another CT-based classification was presented by Bartoníček/Rammelt in 2015 [29]. Five different fracture types were defined: type 1 as an extraincisural fragment with intact fibula notch, type 2 as a posterolateral fragment including the fibula notch, a posteromedial two-part fragment extending to the medial malleolus as type 3 fracture, a posterolateral fragment larger than one-third of the notch as type 4 fracture, and finally irregular osteoporotic fragments as type 5 fracture (Fig. 3). It also includes a treatment algorithm. The Bartoníček/Rammelt classification has been found 46 times in the literature, of these, 21 studies have used it as a classification system, which were included in this study and are shown in Table 5. There is one modification made by Tucek et al*.*, who divided Bartoníček type 4 into three subtypes: subtype 1 as a fracture line that passes laterally past the malleolar groove, subtype 2 as a fracture line that involves the malleolar groove, and subtype 3 as an intercollicular fracture line or a line involving the posterior colliculus [66]. Two studies reported reliability of the classification, both showing substantial interobserver reliability (Fleiss kappa 0.78/Cohen’s kappa 0.744) and almost perfect intraobserver reliability (Fleiss kappa 0.81/Cohen’s kappa 0.936) [24, 32]. Regarding the predictive outcome value, type 1 fractures showed to have better outcome than type 2 fractures [65], and a significantly improved clinical outcome was achieved in type 4 fractures when they were surgically fixed [54]. With increasing fracture type, clinical outcome became worse [1, 21, 63].

**Table 5:** Studies reporting on the Bartoníček/Rammelt classification

Study	Patients/ ankles	Sex/ age	PMF type distribution	Treatment allocation	Assessing Classification	Reliability		Predictive outcome values
						Inter OR	Intra OR	
Bartoníček (2015) [29]	141/141	63m, 78f, 49 (19–83) yo	Type 1 (11), type 2 (74), type 3 (39), type 4 (13), type 5 (4)	Type 1: no fixation; type 4: ORIF, type 2–3: individual decision	Yes	n.r	n.r	n.r
Evers (2015) [47]	42/42	16m, 26f, 52.8 (19–86) yo	Type 1 (2), type 2 (5), type 3 (15), type 4 (2), type 5 (0)—only 24 CTs available	In 16 patients PMFs were fixated by osteosynthesis (75% direct, 25% indirect)	No	n.r	n.r	n.r
Guo (2017) [54]	284/284	156m, 128f, 39.12 (± 12.23) yo	Type 1 (0), type 2 (238), type 3 (5), type 4 (41), type 5 (0)	Type 2: fixation (82) no fixation (156), type 3 were all fixed, type 4: fixation (25) no fixation (16); fixation with AP screws	Yes	n.r	n.r	Type 2: no statistically significant differences in outcome operation vs. no operation; type 4: significantly improved outcome with fixation (AOFAS, VAS)
Yi (2018) [95]	107/107	43m, 64f, 46.58 ± 15.5 yo	Type 1 (1), type 2 (50), type 3 (30), type 4 (26), type 5 (0)	n.r	No	n.r	n.r	PMF in SER group mainly had PL shape, PMF in PER group mainly had PM two-part shape or large PL triangular shape
Zhang (2018) [82]	55/55	33m, 22f, 45.18 (24–83) yo	Type 1 (0), type 2 (9), type 3 (7), type 4 (39), type 5 (0)	Displaced PMF fixated with 1—2 PA screws	Yes	n.r	n.r	Highest rate of PMF violation in type 4, followed by type 3; potency of classification in directing treatment of PMF combined with distal tibial spiral fx confirmed
Bartoníček (2019) [128]	54/54	33m, 21f, 48.2 (19–78) yo	43 of 54 with PMF only 37 CTs, type 1 (5), type 2 (18), type 3 (11), type 4 (3), type 5 (0)	n.r	No	n.r	n.r	n.r
Hendrickx (2019) [129]	164/164	118m, 46f, 41.7 (14–90) yo	36 of 164 with PMF, 2 CTinsufficient: type 1 (0), type 2 (12), type 3 (1), type 4 (21) type 5 (0)	n.r	No	n.r	n.r	n.r
Yang (2020) [136]	27/27	11m, 16f, 61.5 (53–67) yo	Only type 3 (27)	ORIF (combined PL and PM approach)	No	n.r	n.r	n.r
Mertens (2020) [65]	50/50	24m, 26ff, 54 (21–83) yo	Only 46 of 50 patients classified: type 1 (2), type 2 (17), type 3 (13), type 4 (14), type 5 (0)	All ORIF (plate osteosynthesis)	Yes	n.r	n.r	Type 1 show better postoperative results than type 2 (AOFAS score)
Blom (2020) [63]	70/70	30m, 40f, 47 (SD 14.6) yo	Type 1 (16), type 2 (30), type 3 (22), type 4 (2), type 5 (0)	n.r	Yes	n.r	n.r	n.r
Sultan (2020) [75]	369/369	167m, 202f, 46.91 (18–85) yo	247 of 369 with PMF: type 1 (22), type 2 (122), type 3 (54), type 4 (49), type 5 (0)	n.r	No	n.r	n.r	Type 3: highest incidence of intercalary fragment (70%)
Tucek (2021) [66]	19/19	1m, 18f, 58 (20–76) yo	Only type 4 (19)- devided in subtypes 1 (11), 2 (4), 3 (4)	ORIF (PL approach 14x, PM approachl 5x)	Yes	n.r	n.r	Osteoarthritic changes in all four patients with subtype 3, ORIF with good functional results (AOFAS)
Maluta (2021) [1]	46/46	18m, 28f, 55.63 ± 6.11 yo	Type 1 (22), type 2 (18), type 3 (6), type 4 (0), type 5 (0)	Immobilized with cast and no-weight bearing for 5 weeks	Yes	n.r	n.r	Worse outcome with increase of injury severity after 2 years of FU (AOFAS, OMAS)
Neumann (2021) [21]	100/100	31m, 69f, 60.0 (20–83) yo	Type 1 (7), type 2 (35), type 3 (25), type 4 (23), type 5 (0)	No fixation (37), AP screw (14), PA screw (13), plate (36)	Yes	n.r	n.r	No significant postoperative outcome difference between type 1 to 4 (OMAS, FFI, AOFAS score)
Sun (2021) [98]	46/46	20m, 26f, 52.9 ± 14.1 yo	Type 1 (0), type 2 (1), type 3 (36), type 4 (12), type 5 (0)	Single-fragment type: posterior plate fixation with PL approach; double-fragment type: AP screw for PL fragment, PA screw for PM fragment	No	n.r	n.r	n.r
Black (2021) [145]	279/279	93m, 186f, 55 ± 19 yo	Type 1 (20), type 2 (139), type 3 (93), type 4 (24), type 5 (3)	n.r	No	n.r	n.r	n.r
Seo (2022) [148]	153/153	71m, 82f, 48.1 ± 14.6 yo	Type 1 (27), type 2 (81), type 3 (30), type 4 (15), type 5 (0)	ORIF recommended when marginal impaction present, even in small type 2 PMFs	Yes	n.r	n.r	Marginal impaction associated with PMF highly observed in type 2 (58%)
Kleinertz (2022) [32]	113/113	37m, 76f, 56.2 ± 17.8 yo	n.r	n.r	Yes	C.kappa 0.744	C.kappa 0.936	n.r
Patton (2022) [149]	153/153	46m, 107f, 51 ± 8 (18–89) yo	Type 1 (10), type 2 (68), type 3 (44), type 4 (13), type 5 (0), unclassifiable (6)	n.r	No	n.r	n.r	No association to Lauge-Hansen injury mechanism
Pflüger (2022) [24]	193/193	n.r	n.r	n.r	Yes	F.kappa 0.78	F.kappa 0.81	n.r
Sun (2022) [150]	32/32	11m, 21f, 45.6 ± 6.3 (32–59) yo	Only type 4 (32)	PL approach with distal locking plate	Yes	n.r	n.r	n.r

### Mason classification

**Fig. 4:**
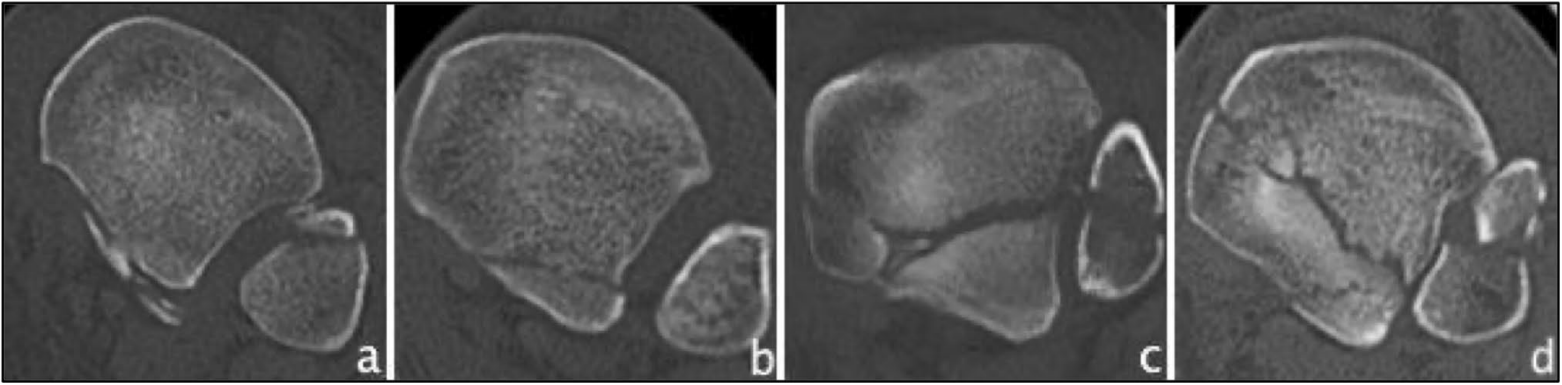
Overview of the Mason classification based on CT images (axial views). **a** Mason type 1 **b** Mason type 2A **c** Mason type 2B **d** Mason type 3

In 2017, Mason et al*.* developed a CT-based classification of PMF ascending in severity of injury [30]. Therefore, Mason described type 1 as an extra-articular avulsion fracture following a rotational force applied to the foot when the ankle is in plantarflexion and the talus unloaded. Rotational forces applied to a loaded foot result in a type 2A fracture in form of a primary triangular posterolateral fragment. A type 2B fracture with a secondary posteromedial fragment, usually angled at 45° to the primary fragment, occurs when the talus continues to rotate in the mortise. A type 3 fracture is characterized by a coronal fracture line that involves the entire posterior plafond due to an axial loading of a plantarflexed talus (Fig. 4). Until now, Mason's classification has been mentioned 22 times in literature, and used for classification in 12 studies, which were included and can be found in Table 6. One modification of Mason type 2B fracture was found. Vosoughi et al*.* divided it into a large intra-articular pilon fragment and a small extra-articular fragment [67]. Interobserver reliability ranged from substantial to almost perfect values (Cohen’s kappa 0.919/Fleiss kappa 0.61/Cohen’s kappa 0.717) as did intraobserver reliability (Fleiss kappa 0.65/Cohen’s kappa 0.957) [24, 30, 32]. As for the predictive outcome value, type 3 fractures tend to show worse postoperative outcome [68].

**Table 6:** Studies reporting on the Mason classification

Study	Patients/ ankles	Sex/ age	PMF type distribution	Treatment allocation	Assesing Classification	Reliability		Predictive outcome values
						Inter OR	Intra OR	
Mason (2017) [30]	121/121	49m, 72f, 48 (17–90) yo	Type 1 (41), type 2A (30), type 2B (25), type 3 (25)	Type 1: no fixation; type 2A: ORIF; type 2B: ORIF, medial fragment first; type 3: ORIF	Yes	C.kappa 0.919	n.r	In type 1 syndesmosis was disrupted in 100% of cases
Jayatilaka (2019) [43]	80/80	n.r., 48.1 (17–90) yo	Only type 2 (33 type 2A, 47 type 2B)	n.r	Yes	n.r	n.r	Rate of syndesmotic injury greater in type 2A than type 2B, although type 2B had greater extent of bone injury
Vosoughi (2019) [67]	47/47	11m, 36f, 46.6 (17–90) yo	Only type 2B	Large pilon intra-articular fragment (29): fixation; small extra-articular avulsion fragment (18): secondary syndesmotic stabilizer	Yes	n.r	n.r	n.r
Mason (2019) [68]	50/50	22m, 28f, 46.8 (21–87) yo	Type 1 (17), type 2A (12), type 2B (10), type 3 (11)	Type 1: syndesmotic fixation; type 2A: ORIF; type 2B: ORIF (medial fragment first) type 3: ORIF	Yes	n.r	n.r	Type 3 fractures tend to worse postoperative outcome (OMAS)
Philpott (2020) [44]	n.r./86	n.r	Only type 2 (2A (33), 2B (34)) and type 3 (19)	ORIF (not homogenous fracture paterns → different appraches)	No	n.r	n.r	n.r
Lee (2020) [134]	166/166	97m, 69f, 44.8 (18–83) yo	PMF in 92 of 166 Fx; type 1 (34), type 2A (18), type 2B (16), type 3 (24)	n.r	No	n.r	n.r	n.r
Yang (2020) [136]	27/27	11m, 16f, 61.5 (53–67) yo	Only type 2B	ORIF (combined posterolateral and posteromedial approach)	No	n.r	n.r	n.r
Gandham (2020) [83]	141/141	54m, 87f, 49.2 (17–90) yo	Type 1 (45), type 2A (41), type 2B (35), type 3 (20)	Type 1: syndesmotic fixation or PL approach; type 2A: PL approach, type 2B: combined MPM and PL approach, PM approach for almost all type 3 fx	Yes	n.r	n.r	n.r
Xie (2021) [28]	108/108	34m, 74f, 49 (18–89) yo	Type 1 (12), type 2A (71), type 2B (22), type 3 (3)	n.r	Yes	n.r	n.r	IAIF was found in more than 2/3 cases of single fragment fractures (type 2A)
Kleinertz (2022) [32]	113/113	37m, 76f, 56.2 ± 17.8 yo	n.r	n.r	Yes	C.kappa 0.717	C.kappa 0.957	n.r
Pflüger (2022) [24]	193/193	n.r	n.r	n.r	Yes	F.kappa 0.61	F.kappa 0.65	n.r
Sun (2022) [150]	32/32	11m, 21f, 45.6 ± 6.3 (32–59)	Type 1 (0), type 2A (10), type 2B (0), type 3 (22)	PL approach with distal locking plate	No	n.r	n.r	n.r

### Quality assessment of included studies

The Coleman score achieved a total median value of 43.5 points (14–79), composed of Part A with a median of 26 points, and Part B with 18 points. Based on the number of patients included, the weighted median total Coleman score was 42.5. Coleman score points are shown in Table 1.

## Discussion

By reviewing the literature, 4 classifications were found describing PMF: a classification based on the fragment proportion in relation to the distal tibial joint surface [45] and the three CT-based classifications according to Haraguchi, Bartoníček/Rammelt, and Mason [14, 29, 30]. The earliest and most commonly used classification was the PMF Classification according to fracture size as first specified by Nelson and Jensen, who postulate a recommendation for treatment of PMF with a fragment size exceeding more than 1/3 of the articular surface on lateral radiographs based on a study sample consisting of 8 patients [45]. With 66 included studies, this classification accounts for the largest proportion of classifications used by surgeons in clinical practice. In the included studies the most used cut-off value was 25%, but also values of 20%, 30% or 1/3 of the articular surface were used.

There are still controversial opinions for osteosynthetic treatment of PMF [69]: McDaniel and Wilson demonstrated, that if a PMF of less than 25% of the tibial joint area was not reduced, it did not significantly affect the overall outcome [58]. De Vries et al*.* and Xu et al*.* found no evidence for fixing PMF smaller than 25%, as outcome scoring systems showed no significant better outcome [52, 53], as well as Guo et al*.* for PMF in tibial spiral fractures [54]. Comparing the outcome of treating PMF less than 25% with that of not fixing it no significant difference in the AOFAS Score was found [55–57]. On the other hand, a trend toward better clinical and radiological outcome in patients in whom PMF was fixed was observed and, therefore, authors recommend PMF fixation of even smaller fragments that cannot be satisfactorily reduced by ligamentotaxis [6, 46, 47, 49, 50]. Baumbach et al*.* and Tosun et al*.* postulated even that in PMF of all sizes, syndesmotic stability is significantly more likely to be restored if treated by open reduction internal fixation [48, 51]. In relation to the total number of studies using this classification, the number of studies in terms of predictive outcome values is rather limited. In the matter of inter- and intraobserver reliability, the available evidence is also meager, Büchler et al*.* were the only ones to study this, providing good results with an inter- and intraobserver reliability of kappa of 0.64 and 0.63, respectively [13]. Of all studies that asses the PMF classification according to fracture size, all but two [6, 49] are of retrospective design. Especially in the earlier studies, the evaluation of the fracture was not optimal, since this was done mainly on the basis of lateral radiographs.

The use of radiographs was found to be limited for the accurate size estimation of PMF [12, 14, 18, 70], therefore, it recently came to the increasing use of computed tomography (CT) in the diagnosis of trimalleolar ankle fractures [18, 47, 54]. Subsequently, the conviction increases that not the size, but the fracture morphology is crucial for the improvement of outcome [19]. Factors such as syndesmotic stability, joint congruity, postoperative step-off, reconstruction of the incisura, intercalary fragments and talar subluxation are thought to be of prognostic importance to consider when treating PMF [7, 23, 48, 50, 51, 53, 58, 63, 71–76]. Hence, a paradigm shift has occurred [21, 24, 31, 77], as also the systematic review by Odak et al*.* has previously shown [22].

This is where the three CT-based classifications come to the fore. The classification used in the majority of studies is the one proposed by Haraguchi [14]. Most probably due to being the first CT-based classification and due to the simple and clear structure dividing the fracture in three types. Since 2015, however, a preference for the Bartoníček/Rammelt classification has emerged, with the main strengths of this classification being the ascending severity of the classification and the derived therapy recommendations [29]. After noting that the Haraguchi classification did not map the mechanism of injury, Mason developed the most recent classification, also considering the injury mechanism [30].

Some objections against Haraguchi’s classification have arisen with the time. First, the classification is not based on severity and thus does not relate to functional outcome [78]. Second, that the classification was based only on axial sectional images and, therefore, fractures were only assessed in one plane, vertical size expansion not being estimated [31], that medial injuries were not evaluated, which may lead to misjudgments [17, 32], and that the extent of involvement of the tibial incisura was not specified, wherefore type I fractures include a wide range of both small and large posterolateral fragments [59]. Most multi-fragmentary fractures cannot be defined using this classification [79]. Also, the three modifications found [61, 62, 80] may suggest that Haraguchi’s classification is not as advanced to represent all fracture types. Regarding the predictive value of the classifications in terms of postoperative outcomes, some authors have shown that type II fractures have worse clinical outcomes [19, 59, 63], whereas Mertens et al*.* observed an improvement in the AOFAS score from type I to type III [65].

The Bartoníček/Rammelt classification was developed on the basis of a larger patient population. It ascends in severity and contains a therapy recommendation [29, 81]. Zhang et al*.* were able to show that the potency of the Bartoníček/Rammelt classification also applies to distal tibial spiral fractures with associated PMF [82]. One objection is the imprecise definition of type 5 fractures, which includes all fractures that cannot be classified as type 1–4. We were not able to find an image of such a type 5 fracture: neither in the original article nor in our own fracture-database. Another objection is the difficulty of estimating 1/3 of the tibial incision to distinguish between a type 2 and type 4 fracture [32]. There is a consistent opinion on worse outcome with increasing fracture type [1, 63, 65]. Only Neumann et al*.* saw an increase in the AOFAS score and no difference in the Olerud and Molander ankle score (OMAS) [21].

The authors of the Mason classification see the advantage in the ascending degree of severity of the classification considering the accident mechanism. They have also introduced treatment recommendations based on their classification. Gandham et al*.* even made a recommendation on the appropriate operative approaches [30, 68, 83]. However, they described the classification using schematic drawings and also do not define the tibial incisura [32]. In addition, a multi-fragmentary fracture of the entire tibial plafond may be mistaken for a two-part posterolateral and posteromedial fracture (type 2B) [32]. With the exception of one study describing a worse outcome in Mason type 3 fractures [68], there are no further statements on predictive values. Until now, Mason’s classification has not yet been able to establish itself in literature with only 12 included studies. In addition, half of all studies using Mason’s classification were conducted by the author's own research group, and it was Mason himself who found the highest interobserver reliability in his study (kappa 0.919) whereas other authors found considerable lower reliability scores (Fleiss kappa 0.61 / Cohen’s kappa 0.717) [24, 30, 32, 43, 44, 67, 68, 83].

Intra- and interobserver reliability are substantial to perfect for all classifications, with Mason scoring the lowest in comparison to the other classifications [24, 32]. However, none of the classifications can adequately describe the complexity of posterior malleolus fracture, as factors such as extent of articular surface impaction, degree of dislocation or intercalary fragments among others are not taken into account [32, 79].

Several important classifications were excluded because they are not PMF-specific. This includes the AO classification originally published in 1987 by Müller/AO, being a universal classification depicting all skeletal injuries. It is a valuable, international classification, which has its justification, and which has been used for years [84, 85]. With the routine use of CT imaging to reliably diagnose and classify trimalleolar fractures [9], authors have shown that all fractures involve the articular surface of the distal tibia [14, 29, 81]. This in contrast to the specification of the AO’s classification through Heim, dividing posterior malleolar fractures into extra- and intra-articular fractures [86]. The AO classification, based on standard plain radiographs, is therefore not suitable for considering the significance and morphology of PMF, nor is it applicable in addressing specific questions regarding PMFs [24, 48, 87].

Classification systems of posterior pilon fractures were also considered to be non-PMF-specific. Hence, the differentiation of pilon fractures from trimalleolar ankle fractures still often causes difficulties in clinical practice [75, 88, 89]. This has led to the emergence of a subset of PMF, also known as the “posterior pilon” variant, which has recently gained popularity [61, 87, 90–93]. However, there is still no clear definition and the understanding of it varies [75, 81, 94, 95]. In addition, there are studies showing that posterior pilon fractures are a separate entity due to morphological differences [61, 94].

Other excluded classifications were sub-entities of PMF fractures. For example, a classification of PMF in tibial shaft fractures (TSF) [96, 97], and one also involving talar subluxation [98].

A few more limitations are worth noting, with majorly the limited quality of the included studies. Limitations affecting the Coleman score include the predominantly retrospective nature of the included studies and small patient cohorts. Therefore, the results of this study could only be presented in a descriptive manner. Only studies written in English were considered, excluding further useful contributions written in other languages.

In conclusion, this review demonstrates that there has been a shift from usage of the PMF classification by fracture size to the newer CT-based classifications, however, none have been able to establish itself in the literature so far. Summarizing all of the previously described points, we believe that, to date, no classification is able to adequately describe the complexity of the PMF. Also, the classifications are weak in terms of a derivable treatment algorithm or prognosis of outcome. According to this review, the Bartoníček/Rammelt classification has the most potential to prevail in the literature and in clinical practice due to its treatment algorithm, its reliability in combination with consistent predictive outcome values.

## Supplementary Information

Below is the link to the electronic supplementary material.

Supplementary file1 (PDF 256 KB)
