# Supplementary material for: Classifications of posterior malleolar fractures: a systematic literature review
Source: Arch Orthop Trauma Surg. 2022 Dec 5;143(7):4181–220. doi: 10.1007/s00402-022-04643-7 (PMC10293398; doi:10.1007/s00402-022-04643-7)
Supplement: Supplementary file 1 — Supplementary file1 (PDF 256 KB) [file 402_2022_4643_MOESM1_ESM.pdf]

Supplementary Figures

Archives of Orthopaedic and Trauma Surgery

Classifications of posterior malleolar fractures:
a systematic literature review

Julia Terstegen[1#], Hanneke Weel[2#], Karl-Heinz Frosch[1,3], Tim Rolvien[1], Carsten Schlickewei[1], Elena Mueller[1]

[1] Department of Trauma and Orthopaedic Surgery, University Medical Center Hamburg-Eppendorf, Hamburg, Germany

[2] Department of Orthopedics, Bergman Clinics, Arnhem, The Netherlands

[3] Department of Trauma Surgery, Orthopaedics, and Sports Traumatology, BG Hospital Hamburg, Hamburg, Germany

# Contributed equally

Corresponding Author:

Elena Müller

Department of Trauma and Orthopaedic Surgery, University Medical Center Hamburg Eppendorf, Martinistr. 52, 20246 Hamburg, Germany.
elena_enderle@hotmail.com, Tel: +49 (0) 40 7410 – 0

Supplementary Figure 1: Modified Coleman Score

Modified Coleman Methodology Score

**Part A – only one score to be given for each of the seven sections**		
1. Study size – number of patients (N)	N > 51	10
	N 31– 50	7
	N 11 – 30	4
	N < 10 or not stated	0
2. Mean follow-up	> 6 years	5
	2.1-6 years	3
	< 2 years, not stated, or unclear	0
3. Percent of patients with follow-up (radiographic and clinical)	> 90%	5
	80-90%	3
	< 80%	0
4. Number of interventions per group	One intervention in all patients in each group	10
	Multiple interventions but consistent among all patients in each group	5
	Unclear, unreported, or multiple interventions among patients in the same group	0
5. Type of study	Randomized control trial	15
	Prospective cohort study	10
	Retrospective cohort study	0
6. Diagnostic certainty (diagnosis confirmed by defined PE findings or MRI)	In all	5
	In $\geq$ 80%	3
	In < 80%, instated, or unclear	0
7. Description of surgical technique	Technique stated with necessary details to repeat	5
	Technique named without elaboration	3
	Not stated or unclear	0
8. Description of postoperative rehabilitation	Well described with $\geq$ 80% patient compliance	5
	Well described with 60–80% patient compliance, or described without complete detail	3
	Protocol not reported < 60% patient compliance	0
**Part B – scores may be given for each option in each of the three sections if applicable**		
1. Outcome criteria	Outcome measures clearly defined	2
	Timing of outcome assessment clear	2
	Use of outcome criteria with reported good reliability	3
	Use of outcome with good sensitivity	3
2. Procedure for assessing outcomes	Subjects recruited	5
	Independent investigator (2 for radiographic, 2 for clinical)	4
	Written assessment	3
	Patient centered data collected	3
3. Description of subject selection process	Selection criteria reported and unbiased	5
	Recruitment rate reported and $\geq$ 80 %	5
	Eligible subjects not included in the study satisfactorily accounted for	5
	Total	100
